# 

**Published:** 1885-09

**Authors:** 


### CONTENTS:

Art. I—Address, By Edward N. Harris, D. D. S., of Boston........................193
Art. II—Rigg's Disease, By G. A Mills........................................218
Art. III—A German Trial against Dentists for using the title "Doctor of Dental Surgery," Translated by George C. Gaston..........................223

National Association of Dental Examiners.....................................228

### EDITORIAL, ETC.

The National Association of College Faculties................................230

### OBITUARY.

Dr. Isaiah Forbes............................................................232

### MONTHLY SUMMARY.

Fracture of Jaw and Treatment................................................233

The International Medical Congress...........................................235

Saving Roots.................................................................236

Ancient Teeth................................................................237

The Most Popular Medicines...................................................238

Creosote in Dentition........................................................239

For Indigestion..............................................................239

An Old Bullet................................................................239

Reflex Pain..................................................................240

